# D-Penicillamine: The State of the Art in Humans and in Dogs from a Pharmacological and Regulatory Perspective

**DOI:** 10.3390/antibiotics10060648

**Published:** 2021-05-28

**Authors:** Michela Pugliese, Vito Biondi, Enrico Gugliandolo, Patrizia Licata, Alessio Filippo Peritore, Rosalia Crupi, Annamaria Passantino

**Affiliations:** 1 Department of Veterinary Science, University of Messina, 98166 Messina, Italy; mpugliese@unime.it (M.P.); vbiondi@unime.it (V.B.); egugliandolo@unime.it (E.G.); plicata@unime.it (P.L.); passanna@unime.it (A.P.); 2 Department of Chemical, Biological, Pharmaceutical, and Environmental Science, University of Messina, 98168 Messina, Italy; aperitore@unime.it

**Keywords:** D-penicillamine, humans, dog, copper-associated hepatitis, prescription

## Abstract

Chelant agents are the mainstay of treatment in copper-associated hepatitis in humans, where D-penicillamine is the chelant agent of first choice. In veterinary medicine, the use of D-penicillamine has increased with the recent recognition of copper-associated hepatopathies that occur in several breeds of dogs. Although the different regulatory authorities in the world (United States Food and Drugs Administration—U.S. FDA, European Medicines Agency—EMEA, etc.) do not approve D-penicillamine for use in dogs, it has been used to treat copper-associated hepatitis in dogs since the 1970s, and is prescribed legally by veterinarians as an extra-label drug to treat this disease and alleviate suffering. The present study aims to: (a) address the pharmacological features; (b) outline the clinical scenario underlying the increased interest in D-penicillamine by overviewing the evolution of its main therapeutic goals in humans and dogs; and finally, (c) provide a discussion on its use and prescription in veterinary medicine from a regulatory perspective.

## 1. Introduction

Despite its name’s similarity to the widely known antibiotic penicillin, D-penicillamine (DPA) is a byproduct of penicillin without any antibiotic properties [1]. It is a known chelating agent, a chemical compound used to trap or remove heavy metals, such as copper, lead, iron, and mercury, from the body; moreover, DPA also showed positive immunomodulatory and antifibrotic capacities [2,3,4,5].

**Figure 1:**
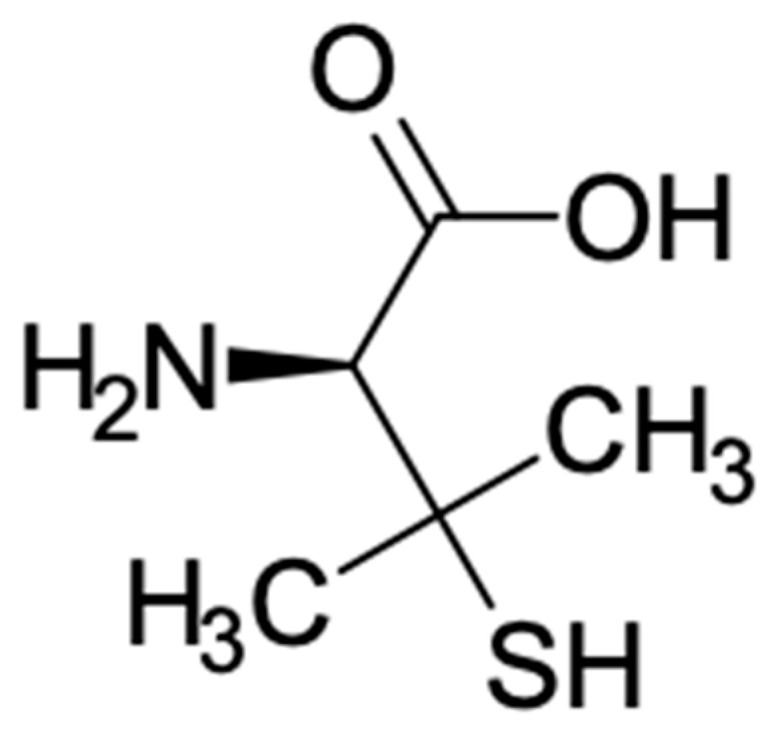
Structure of D-penicillamine.

Penicillamine, also named D-b, b-dimethylcysteine, was firstly classified as a degradation product of penicillin [6], and chemically is a structural analog of cysteine, with two methyl groups in place of the two hydrogen atoms linked to the second carbon atom of cysteine [7]. For its structure, penicillamine shows similar properties to cysteine (Cys) and, for this reason, it is classified as a nonproteinogenic amino acid containing a thiol group [8]. Penicillamine is also considered a trifunctional amino acid, in which an amino group and a carboxyl group are attached to one carbon atom, and a sulfhydryl and two methyl groups to a second (Figure 1) [7]. The three functional groups in penicillamine undergo characteristic chemical reactions and change in their ability to participate in acid–base equilibria, nucleophilic addition and displacement, combination with various metals, oxidation, and free radical transformations [9]. Two are the enantiomers recognized: D and L isomers. In particular, D isomer is the only one that can be utilized in clinical practice, although the L isomer is characterized by excessive toxicity [1].

In humans, DPA, due to its metal chelating properties, is used to treat several diseases, including Wilson’s disease [10], heavy metal intoxication [11], cystinuria [12], and rheumatoid arthritis [13]; at the same time, DPA’s chelating ability is also used in veterinary medicine, and it is used in the treatment of liver disease caused by the accumulation of abnormal storage of copper [14]. In fact, it is considered the initial treatment of choice for most dogs with copper-associated hepatitis, including those with clinical illness and those with moderate to severe hepatic histopathologic abnormalities [15]. Furthermore, this veterinary medicine is also used for the long-term oral treatment of lead, or cadmium, and mercury poisoning, or cysteine urolithiasis [16,17].

Though DPA remains an effective drug for many dogs with copper-associated hepatitis, there is a limitation to its use due to scarce availability for the reason that it is marketed only for humane use [14].

This lack of animal equivalent product results in veterinarians using this drug outside of the authorized conditions of use, detailed in its summary of product characteristics to treat disease and alleviate suffering [14,18,19,20].

Given that DPA has increased in recent years for its effectiveness in the treatment of copper-associated hepatopathies, decreasing hepatic copper concentration and diminishing the grade of inflammation, the authors: (a) address the pharmacological features; (b) outline the clinical scenario underlying the increased interest on DPA, by overviewing the evolution of its main therapeutic goals in humans and dogs; and finally, (c) provide a discussion on its use and prescription in veterinary medicine from a regulatory perspective.

## 2. Absorption, Distribution, Metabolism and Excretion (ADME)

Penicillamine is absorbed rapidly from the gastrointestinal tract, with an oral bioavailability between 40 and 70%. Literature studies show that its availability is significantly reduced when taken with iron supplements, antacids, or food; in fact, peak concentrations in blood are achieved in 1–3 h after administration [21]. 

In contrast to cysteine, penicillamine is quite resistant to attacks by cysteine deoldhydrase or L-amino acid oxidase, so it is stable in vivo. Almost all penicillamine is degraded by the liver and its metabolites have been identified in both urine and feces [22]. Once penicillamine is ingested, it transforms into disulfides [21,22], inorganic sulphates, N-acetyl-D-penicillamine, and S-methyl-D-penicillamine [23]. 

Among metabolites, the disulfides are the most important, since they bind to albumin, and this binding is responsible for the slow elimination of the drug from plasma [24]. It was demonstrated that 80% of penicillamine in plasma is protein bound, whereas 6 % is found in its free form, and the remaining metabolites account for 14 % [25]. Moreover, s-methyl-D-penicillamine is further oxidized into sulfoxide or sulfone. Patients who are poor sulfoxidizers have demonstrated an increased rate of immunologically mediated toxicity from penicillamine [26]. It was suggested that penicillamine is slowly released from deep tissues and skin [27]. Penicillamine is considered a chelating agent for lead, copper, iron, and mercury [16,17,28].

Moreover, penicillamine also acts as an anti-inflammatory agent [29]. It inhibits collagen cross-linking by making it more susceptible to enzyme degradation [30]. The antifibrotic properties reported may be useful in the treatment of animals with hepatitis, but more data are necessary to validate this aspect.

### Mechanism of Action

Structurally penicillamine is related to L-cysteine, an amino acid which is normally present in the body. Penicillamine has a long list of biological actions and is naturally produced by degradation of the thiazolidine ring after cleavage of the β-lactam ring in penicillin [31]. The metal-binding ability of DPA is believed to underlie its effectiveness in the treatment of Wilson’s disease, an inherited disorder of copper metabolism. DPA is thought to decrease excess copper levels in Wilson’s disease by reducing Cu(II) to Cu(I) [32]. The reduction is accompanied by a change in preferred geometry from square planar to tetrahedral, and a change in net charge, both of which are less favorable for protein binding. Although EDTA binds copper with equal or greater affinity, it is thought to have a lower efficacy than DPA in the treatment of Wilson’s disease due to its inability to reductively chelate copper. 

DPA has also been used for 30 years to treat rheumatoid arthritis, but its mechanism of action remains unknown [33]. The current understanding of metalloprotease involvement in the pathogenesis of arthritis suggests that the efficacy of DPA may be explained by the inhibition of zinc proteases involved in remodeling the extracellular matrix. Because DPA can chelate zinc with its thiol, amino, or carboxylate groups, it is tested for the inhibition of three zinc proteases, matrilysin, thermolysis, and carboxypeptidase A, since these enzymes represent the matrix metalloprotease, metalloendoprotease, and metalloexoprotease families, respectively [31]. However, it is still not known what the mechanism of action is in rheumatoid arthritis or in rheumatic diseases in general. There are certainly several ways to go, i.e., Jaffe et al. [34] used their knowledge about the potential of penicillamine to split the IgM rheumatoid factor molecule as rationale for the introduction of penicillamine in the treatment of rheumatoid arthritis.

In certain experimental models, DPA inhibits humoral and/or cell-mediated immune reactions, as well as particular inflammatory reactions, such as prostaglandin synthesis modulation, lysosomal enzyme release, and oxyradical generation [35]. DPA inhibits lysyl oxidase, which deaminates the lysine to provide polypeptide cross-links in elastic fibers and collagen fibers [36,37]. DPA may directly bind to immature forms of collagen and elastin, preventing formation of mature cross-links, or it may interfere with copper-dependent enzymes responsible for these cross-linking reactions [38]. DPA inhibits the zinc proteases involved in remodeling of the extracellular matrix [36]. It may act at the site of inflammation by impairing fibroblast proliferation [39]. It also regulates histamine activity by potentiating its metabolic inactivation, which may be the cause of its antipruritic effect in patients [35].

## 3. Clinical Applications in Humans and Companion Animals

### 3.1. In Humans 

The United States Food and Drugs Administration (FDA) approved penicillamine for the treatment of several diseases, like Wilson’s disease, rheumatoid arthritis, and cystinuria. Owing to its toxicity, penicillamine was discontinued in rheumatoid arthritis and is confined to patients with an extreme active disorder, and who failed to respond to an effective traditional treatment. Penicillamine also presented some off-label applications among others, including lead poisoning [8], scleroderma [40], biliary primary cirrhosis [41], and retinopathy of prematurity [42,43], as reported in Table 1.

An important cause of death in RA patients is secondary amyloidosis, in addition to kidney disease; a potential use of DPA as a treatment for secondary amyloidosis has been hypothesized in the first few years [44,45].

Thanks to the different modes of action proposed for DPA, such as eliminating free radicals from oxygen or facilitating the synthesis of heme and protecting the peroxidation of biomembranes by enhancing the action of antioxidant enzymes containing heme, DPA has been proposed as a treatment off-label in various pathologies [46,47]. Among the various off-label uses of DPA it has also been proposed in retinopathy of prematurity, where it has been tolerated and has no major short-term adverse effects [43].

Much research on the utility of penicillamine as an anti-cancer agent is being gathered. For example, an in vitro study on lung and oral cancer, penicillamine showed a potent protector activity against cigarette smoke. Penicillamine has anti-aldehyde and anti-inflammatory qualities that inhibit redox reactions between tobacco smoke and mucus [29]. 

Penicillamine may also play a role in treating Alzheimer’s patients due to its anti-oxidant effects, although more studies are required [48].

The antiangiogenic properties of penicillamine are also shown, as they can inhibit many significant growth factors (for example, vascular endothelial growth factor and fibroblast growth factor) needing copper as a cofactor [49].

Due to its ability to bind copper, DPA has also been proposed as a potential treatment for hepatitis; in fact, it was seen in a model of hepatitis on Long-Evans Cinnamon rats how DPA reduced copper levels in the liver compared to animals not treated [50].

In cancer patients, higher levels of copper and oxidative stress than in healthier people are well known [51]. The oral administration of penicillamine lowers copper and ceruloplasmin in those patients in the phase II clinical study on antiangiogenic involvement of penicillamine in glioblastoma [52]. Furthermore, DPA due to its ability to act on acetaldehyde, implicated in the damaging mechanism of ethanol, has been used for the treatment of ethanol-induced conditioned place preference, a behavioral disorder related to alcohol intake [53].

#### 3.1.1. D-Penicillamine in Wilson Disease and Metal Accumulation

DPA was proposed in 1956 as the first oral drug, and second overall drug, for the treatment of WD, thanks to its high affinity to chelate copper not bound to ceruloplasmin at the plasma level [58]. Penicillamine is a chelant of copper and other divalent ions, such as cadmium, lead, mercury, and nickel [59]. Although, elevated levels of copper have been reported in serum and synovial fluid [60] of patients with rheumatoid arthritis. After prolonged administration of DPA to rheumatoid patients, the titre of rheumatoid factor decreases [61]. The leading cause of chronic copper intoxication today is Wilson’s disease, traditionally treated by chelation with DPA. In the long-term treatment of metal storage diseases, DPA chelation therapy has played a crucial role; mercury poisoning, and lead and copper poisoning had previously been treated with chelators, such as DPA [28]. Over the years, new chelants have been introduced that have the same affinity, but lower toxicity than DPA, such as meso-2,3-dimercaptosuccinic acid (DMSA) and 2,3-dimercapto-propane sulfonate (DMPS), capable of effectively mobilizing mercury and lead deposits in the urine. DMSA was more efficient than DPA in reducing tissue levels and increasing urinary excretion of lead in mice and rats, as compared to the long-term therapeutic effects of DPA [62]. DMSA appeared superior in that it caused clinical symptoms to exacerbate less frequently than DPA [63].

Thanks to its copper chelating effect, DPA has also been proposed as a treatment in patients with chronic liver disease, in which a low dose has been noted have positive effect on preterminal Indian childhood cirrhosis, which was shown to be associated with long-term survival [64].

Copper normally accumulates in the liver of patients with the primary biliary cirrhosis syndrome, and the amounts can equal or surpass those recorded in Wilson’s disease [65]. Copper will be mobilized from the body and the hepatic copper content will be reduced with DPA and a low copper intake [66]. In the past, DPA, due to its antifibrogenic, cupruretic, and immunosuppressive effects, has also been suggested as a potential treatment for primary sclerosing cholangitis (PSC), a chronic hepatobiliary disease characterized by diffuse inflammation and fibrosis of the leading intrahepatic and extrahepatic bile ducts at hepatic overload of copper. Despite the high expectations, DPA has not shown positive effects in the treatment of PSC and is, therefore, not recommended for any further studies due to poor efficacy, accompanied by frequent serious side effects [67].

#### 3.1.2. Neonatal Period

In the early 1970s, the scientists reviewed the role of DPA in the treatment of NHBI (neonatal hyperbilirubinemia) [68], a new drug for most of neonatologists. The idea that DPA might be a suitable drug to act as a copper-binding agent to control icterus neonatorum occurred, serendipitously, while reflecting on the similarity of copper storage in Wilson’ s disease and neonates [69]. It is well known that all neonates have elevated copper levels in the liver and reduced plasma copper protein concentrations, ceruloplasmin, relative to adults over age one [70]. 

In the premature and term babies 4–6 h after administration, the effect of a single intravenous injection of 100 mg/kg body weight of DPA on SEBI (serum bilirubin concentration) can be observed. A sudden decline in SEBI occurred only in term infants with elevated SEBI, but DPA does not affect infants under 1500 g in birth weight (suffering from so-called accumulating NHBI by the immaturity of the glucanosyltransferase enzyme system) or terminal infants with low SEBI. A plausible explanation for this is that DPA inhibits bilirubin formation but does not cause any change in UDP glucanosyltransferase activity [71]. 

#### 3.1.3. Dermatological Application and Cutaneous Adverse Effects

After the examination of its cutaneous effects in Wilson’s disease patients, penicillamine was first proposed as a possible therapeutic agent in systemic sclerosis (SSc) with reduced skin collagen and general skin thinning [27]. In vitro experiments have shown that the development of intra- and inter-molecular collagen crosslinks is interfered by penicillamine, which contributes to the tissue aggregation of unrelated collagen molecules which are most susceptible to proteolytic enzymes [31]. Several uncontrolled SSc studies found that penicillamine improved the skin sclerosis condition, lowered the rate of involvement of the new visceral organ and improved overall survival [8]. In a prospective study conducted for 15 years, 69 patients with rapidly progressive SSc received penicillamine at a dosage of 750 mg/day for at least 6 months [72]. Penicillamine treatment strengthened defences to skin sclerosis lesion and simultaneously arrested pulmonary involvement. In high dose penicillin therapy, however, 80% of the subsequent withdrawal occurred.

Moreover, numerous studies have demonstrated the utility of penicillamine in localized scleroderma care [73]. 

By preventing dopachrome production, penicillamine presented a melanogenesis inhibition influence [74]. Although the nonpigmented melanoma cells are mostly susceptible to gamma radiation, it is used in vitro as a radiation sensitizer to destroy the melanoma cells that inhibit the penicillamine [75].

Furthermore, there is evidence to show the therapeutic efficacy of penicillamine in eosinophilic fasciitis patients (EF) [76]. EF is a rare condition with associated eosinophilia characterized by symmetrical thickness and skin hardening, especially on the forearms and the thorax. While corticosteroids are the first treatment line, certain patients do not react and/or can have serious side effects on long-term treatment. Although no controlled studies are necessary for EF patients on the effects of penicillamine, some findings indicate a beneficial impact, including on patients that are not corticosteroid tolerant, often leading to a total remission [77,78,79,80,81]. Keloids, which are symptoms of intensified collagen production, are one of the disorders for which DPA is used as a medication [82]. Collagen cross-linking is prevented by agents like b-amino propionitrile (BAPN) and DPA, making it more vulnerable to enzymatic degradation [83]. Since BAPN has little effect on collagen that has already been cross-linked, and DPA works at a separate site, the two should be used together. In patients who are known to develop keloid scars, topical application of BAPN and DPA can help prevent the formation of keloid scars [82]. Lipid proteinosis appears to be a consequence of a specific overproduction of type IV collagen of the basement membrane by epithelial or endothelial cells, and of an increased synthesis of non-collagen glycoproteins by fibroblasts [84]. Given the ability of DPA to bind directly to immature forms of collagen and elastin, preventing bonding or interfering with the copper-dependent enzymes responsible for these cross-linking reactions it is considered a promising agent for the treatment of lipoid proteinosis, especially when used in low doses [35].

### 3.2. In Animals

Through its immunomodulatory and antifibrotic properties, DPA has shown to be effective in the treatment of copper hepatitis in dogs, in which it is used as a potent chelant agent. It is administrated at a dose of 10–15 mg/kg orally twice daily [85,86,87].

Inherited copper-associated hepatitis in dogs is a copper storage disorder similar to Wilson’s disease described in humans, characterized by an abnormal hepatic copper accumulation. The disease has been identified in different breeds including the Bedlington terrier, West Highland white terrier, Skye terrier, Dalmatian, Dobermann, and Labrador retrievers [88,89,90,91,92,93]. The pathogenesis is partially known. In Bedlington terriers a genetic predisposition for deletion of exon 2 of the COMMD1 gene is present, causing an extreme accumulation of copper in the liver [94,95,96], while, in the other breeds, where copper concentration is not very high as in the Bedlington terrier, it seems that environmental factors play an important role in the pathogenesis of hepatic copper accumulation [96]. A higher incidence is recorded in females than males, and in Labrador retriever the disease seems to show with more clinical signs, where the continuous copper accumulation in the liver causes hepatitis and, in the end-stage of liver disease, the onset of cirrhosis precludes a successful treatment. Supplementation on a diet of zinc salt is used to create a negative copper balance by blocking the copper adsorption into the enterocytes [97]. The disease is characterized by an early phase, during which the copper is stored in the liver without clinical signs. In the clinical phase, dogs show clinical findings related to the liver dysfunction, such as polyuria, polydipsia, vomiting, anorexia, diarrhea, lethargy, icterus, ascites, and convulsions. 

Usually, the diagnosis was performed in this phase, representing the several-stage of liver disease associated with fatal progression within a few months. The diagnosis of copper hepatitis is histological on liver biopsies, necessary for managing the progression of the disease and for monitoring the improvement post-therapy. 

The liver biopsy verifies the diagnosis of copper-associated hepatitis coupled with hepatic copper concentrations greater than 2000 mg/g [98], and with monitoring of the clinical status (presence or absence of appetite, presence of vomiting and/or diarrhea, degree of polydipsia, abnormal behavior, etc.), which is paramount when using DPA. It should be appropriate also to carry out a physical examination, especially of the liver (palpation of its margins to evaluate the presence of enlargement, nodules, or pain).

Treatment with copper chelators like DPA is efficacious in dogs in the asymptomatic phase, where the diagnosis is usually performed during a screening of family members. The treatment aims to create a negative copper balance, using copper chelant as DPA, stimulating the cupriuresis [14,99,100].

## 4. Adverse Effect in Humans and Animals

### 4.1. In Humans

Unfortunately, the beneficial effects of penicillamine may be accompanied by a variety of undesirable side effects.

Adverse reactions for DPA leading to withdrawal included rash, nausea and vomiting, thrombocytopenia, and proteinuria, all responding to withdrawal of the drug. In a previous study, an incidence of adverse reactions to DPA was seen in 155 patients with rheumatoid arthritis that was analysed and compared with their history of adverse reactions to gold. Patients who have adverse reactions to gold are significantly more likely to develop side effects from DPA [101]. 

The first oral drug for the treatment of WD was DPA [102]; in fact, its efficacy on liver diseases has been demonstrated in several studies [103,104,105]. Serious adverse side effects have been associated with treatment with DPA, linked to nephrotoxicity, bone marrow toxicity, skin elastosis, and others, posing a huge question on the safety profile of the latter [106,107]. Even at the pediatric level, the main treatment for WD involves the use of copper and zinc chelators [108,109]. Due to the side effects over the years, the combined use of different chelators, such as penicillamine and trientine, has been considered, which, despite showing many positive effects, also has a series of toxicological effects. 

In particular, it was seen how the use of copper chelators in patients who developed neurological complications progressively required an interruption of therapy [110]. It was also found in a previous study that approximately 10% of WD patients reported neurological deterioration [111,112,113]. The mechanism underlying the neurological deterioration in WD patients by copper chelation with DPA treatment is not very clear. One hypothesis of this neurological worsening could be that DPA leads to a mobilization of the copper present in the liver to the brain via the blood [114,115]. 

It has been seen in several clinical cases how DPA treatment was capable of developing autoimmune phenomena [116,117]. For example, in some clinical studies on rheumatoid arthritis, WD, or cystinuria, treatment with DPA caused the development of autoimmune diseases, symptoms that regressed following the discontinuation of treatment with DPA [118].

DPA was able to chelate heavy metals, so may bond the gold stored in tissue for prolonged periods after the end of treatment. Hence, some adverse reactions to DPA seen in patients after treatment with gold may result from mobilisation of gold in the tissues. The frequency and severity of adverse reactions can be reduced by the gradual introduction of DPA therapy and the administration of low maintenance doses [119].

DPA haematological toxicity is close to adverse effects in experimental therapies, as seen in reduced platelets and white blood cells, and seems to not be dose-related [120]. Despite the continuity of treatment, depressions within hematopoietic cell counts are intermittent and usually resolved. The propensity of DPA to chelate copper and other basic minerals or short-term cytotoxicity due to its metabolites are theoretical causes of such temporary impact [101].

The leukocyte and platelet count, and urinary analyses for proteinuria and haematuria must be repeated throughout the treatment period at monthly intervals and, in the first eight weeks of therapy, these tests must be performed at weekly or biweekly intervals. A drop in the number of leukocytes and platelets in the three subsequent counts suggests an interruption of therapy, even if the values remain within normal limits. Proteinuria should be measured over 24 h. A gradually progressive increase in proteinuria or the appearance of significant haematuria are reasons for discontinuing therapy.

The penicillamine treatment presents cutaneous manifestations very different that may be divided into several categories, such as the effects on collagen and elastin, autoimmune nature effects, acute sensitivity reactions, and a variety of miscellaneous side effects that have not been included in the others categories [121]. 

Dermatological responses are usually the development of hives, but often macular or popular [9]. Medicinal eruptions typically occur within 7 to 10 days of initiation, and clear within a few days of discontinuation [9]. 

An early onset allergy, unless severe, responds to treatment with cyproheptadine and temporary reduction of the dose of DPA.

Most of these lesions can be explained on the basis of an immune mechanism or a toxic-metabolic effect on connective tissue synthesis. The concept of the interaction of a disease entity with a specific drug and the subsequent production of a variety of adverse effects based on dosage and duration of therapy is fascinating.

### 4.2. In Animals 

In dogs side effects are usually referred to as gastrointestinal signs, such as vomiting, anorexia and, diarrhea [122]. DPA administration with food is generally recommended to avoid or reduce vomiting in dogs treated [93,122]; despite similarity to what happens in humans, its biodisponibility with the food decrease is about 70% [122]. The occurrence of vomiting at approximately the time of peak plasma drug concentration (1–3 h), suggests that the onset of gastrointestinal side effects is related to blood concentration and to stimulation of the chemoreceptor trigger zone [123]. In order to reduce the vomiting a long-acting antiemetic could be administered one hour before DPA administration. 

Other side effects reported in human medicine, are not described in dogs. In the literature, two cases are documented with the suspicion of immunological side effects following DPA administration. A female English springer spaniel showed ascites and proteinuria after four months of treatment with DPA, relating to the presence of protein-losing glomerulopathy. After the cessation of treatment, a resolution of clinical signs was detected [122]. Another case was a West Highland white terrier with several dermatological lesions, rapidly and totally solved after the interruption of the treatment with DPA [122]. The DPA may cause a decrease in the excretion and on the activity of pyridoxine (B6 vitamin). Pyridoxine supplementation should be considered in the long-term treatment with penicillamine [124]. 

## 5. Prescription in Veterinary Medicine from a Regulatory Perspective

Veterinarians are often faced with cases for which approved drugs are not available for the complete range of animal species and disease conditions encountered, or where extra-label use may be more effective or appropriate [125].

In the present case, DPA is registered for use in humans only, but veterinarians often prescribe it for animals as an “extra-label” (used in a manner not in accordance with approved labeling) drug. When drugs such as this are rarely available, veterinarians are granted the privilege of using human drugs in an off-label way.

### Extra-Label Drug Use (ELU)

ELU refers to the use of a pharmaceutical product in a manner that is not consistent with what is indicated on the label, package insert, or product monograph of any drug product approved by a regulatory authority [126]. 

ELU also refers to: (*i*) any approved drug that is administered in a manner not explicitly stated on the approved label regarding the indication, dosage regimen, route (oral *versus* injectable) or frequency of administration, duration of treatment, target species (i.e., dogs instead bovine), or age groups (i.e., puppies instead of adults) [127]; and (*ii*) any drug approved for humans but not for the veterinary use [127].

Therefore, when an unapproved drug is used in a manner that has never been approved, it is referred to as an off-label use.

The rationale for ELU is that the official agencies do not regulate the practice of drugs, and that veterinarians/physicians are free to decide what they consider best for their patients.

## 6. Discussion

Although the pharmaceutical industry has grown progressively over the years and a variety of veterinary pharmaceutical agents are marked [128,129], there remains many human drugs that are prescribed extra-label for animals when there is not an animal equivalent drug product available, as in the case of DPA, in order “*to avoid causing unacceptable suffering*” (art. 112 of the Regulation 2019/6/UE on Veterinary Medicinal products). In these cases, it is recognized that ELU is necessary and appropriate. The use of the expression, *in particular, to avoid causing unacceptable suffering* allows the legislator to indicate that ELU is restricted. The choice to use this chelant agent extra-label is made by the prescribing veterinarian under his/her direct personal responsibility because this use might be associated with adverse events [130,131].

Veterinarians, playing a pivotal role in animal health and welfare, must ensure that they meet the practice expectations when prescribing and dispensing a drug in an extra-label manner [132], as reported in Box 1.

Box 1 **Practice Expectations of the Veterinarian.**

1. Obtain an informed consent from the owner when prescribing a drug in an extra-label manner as suggested in humans for the physicians [133]

2. Understands that he/she has the responsibility to ensure the safety, efficacy, and, in the case of therapy in food-producing animals, the food safety when prescribing an extra-label drug use [134,135,136].

3. Recommends a drug approved for veterinary use as the first drug treatment option where available. Alternatively, recommends a drug approved for human use. When a not approved drug exists and where a therapeutic need has been established, recommends that a drug be compounded from a drug approved for veterinary use, or a drug approved for human use, or (if neither is possible) from an active pharmaceutical ingredient.

4. Understands that prescribing a compounded product instead of an approved drug should not be done solely for the economic benefit of the veterinarian.

5. Prescribes in an extra-label manner in keeping with the current research and evidence for a specific species.

Thus, veterinarians are expected to use their professional judgement to determine the appropriateness of ELU in individual patients, although a not explicit guidance in exercising such judgement is available [137]. 

**Figure 2:**
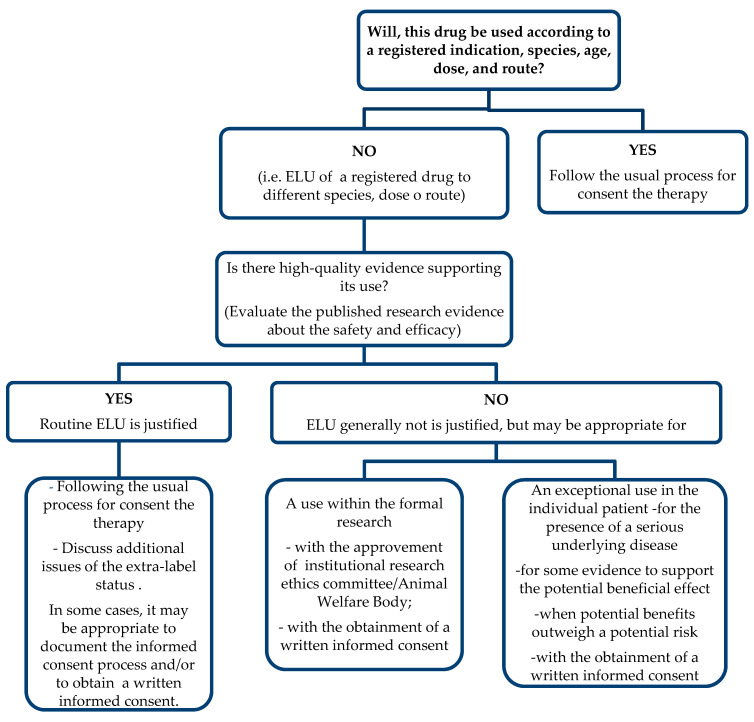
Evaluating the appropriateness of extra-label use (ELU) in dogs.

To provide a systematic process for assessing the appropriateness of any proposed ELU, a decision tree/algorithm (Figure 2) with accompanying explanatory notes should be developed. The notes guide the veterinarian considering ELU of a particular drug, such as DPA, in answering the question: “Is there high-quality evidence supporting its use?” The answer derives from a critical evaluation of the best available scientific evidence based on the efficacy and the safety.

Routine ELU of DPA in the dog could be justified because there is sufficient evidence supporting efficacy of this molecule [82,118,119], suggesting an overall reasonable benefit–risk ratio, given the severity of the clinical condition due to the copper hepatitis.

Unfortunately, few studies relating to the pharmacokinetics of DPA in dogs exist [122,138,139]; therefore, the rationale for using this product is often based on data from human studies, along with empirical clinical veterinary experience.

## 7. Conclusions

It could be appropriate to approve DPA that already exists on the human drug side with an animal-approved label claim. In this manner, it might assure good quality care for dogs with copper-associated hepatitis. Further studies on the pharmacology, pharmacodynamic, and efficacy of DPA in dogs are needed.

## Figures and Tables

**Table 1:** Penicillamine: therapeutic uses.

US FDA Approved	References	Off-Label Uses	References
Rheumatoid arthritis	[33]	Lead poisoning	[8]
Wilson disease	[32]	Retinopathy of prematurity	[42,43]
		Primary biliary cirrhosis	[41]
		Keloids	[54]
		Hemophilic synovitis	[55]
		Lipoid proteinosis	[35]
		Amyloidosis	[45]
		Primary sclerosing cholangitis	[56]
		Chronic active hepatitis	[25]
		Alcohol detoxification	[25]
		Keratosis follicularis	[57]
		Systemic sclerosis (SSc)	[27]

## Data Availability

Data available on request.
